# Dexketoprofen-Loaded Alginate-Grafted Poly(N-vinylcaprolactam)-Based Hydrogel for Wound Healing

**DOI:** 10.3390/ijms26073051

**Published:** 2025-03-26

**Authors:** Tudor Bibire, Alina-Diana Panainte, Catalina Natalia Yilmaz, Daniel Vasile Timofte, Radu Dănilă, Nela Bibire, Larisa Păduraru, Cristina Mihaela Ghiciuc

**Affiliations:** 1 Department of Surgery, Faculty of Medicine, “Grigore T. Popa” University of Medicine and Pharmacy from Iasi, 16th Universitatii Street, 700116 Iasi, Romania; tudor_cd_bibire@d.umfiasi.ro (T.B.); radu.danila@umfiasi.ro (R.D.); 2 “St. Spiridon” County Clinical Emergency Hospital, 1st Independentei Blvd., 700111 Iasi, Romania; 3 Department of Analitical Chemistry, Faculty of Pharmacy, “Grigore T. Popa” University of Medicine and Pharmacy from Iasi, 16th Universitatii Street, 700116 Iasi, Romania; nela.bibire@umfiasi.ro (N.B.); larisa-paduraru@umfiasi.ro (L.P.); 4 Biochemistry Division, Department of Chemistry, Faculty of Science, Dokuz Eylül University, Buca, Izmir 35390, Turkey; catalinanatalia.yilmaz@deu.edu.tr; 5 Clinical Pharmacology and Algeziology, Department of Pharmacology, Faculty of Medicine, “Grigore T. Popa” University of Medicine and Pharmacy from Iasi, 16th Universitatii Street, 700116 Iasi, Romania; cristina.ghiciuc@umfiasi.ro; 6 “St. Maria” Clinical Emergency Hospital for Children, 62nd Vasile Lupu Street, 700309 Iasi, Romania

**Keywords:** sodium alginate, poly(N-vinylcaprolactam), dexketoprofen, graft copolymerization, thermo-responsive matrix, wound healing

## Abstract

All acute and chronic wound management strategies have limitations. Therefore, there is an urgent need to develop new treatment options for wound healing. Hydrogels based on natural polymers offer advantages in wound management because they can reduce patients’ pain, fight infection, and carry targeted drugs to speed up the healing process. In this study, we aimed to develop and investigate an alginate-grafted N-vinylcaprolactam-based matrix for a modified release of dexketoprofen (DEX), which is potentially useful in wound healing. Free radical polymerization and grafted techniques were used to prepare thermo-responsive hydrogels. The obtained hydrogels, unloaded hydrogel (HY) and dexketoprofen-loaded hydrogel (DEXHY), were characterized and analyzed. The concentration of DEX encapsulated in the polymer matrix was 4 mg/mL. The IC50 values found for the samples tested by us were 607.4 µg/mL for HY, 950.4 µg/mL for DEXHY, and 2239 µg/mL for DEX. The average value of cell viability (%) after the exposure of cells to DEXHY hydrogel was 75.4%. DEXHY exhibited a very good in vitro wound closure rate, given its ability to modify DEX release kinetics. The hydrogel developed in this study has shown considerable potential to facilitate and even accelerate wound healing, including surgical wounds, by inhibiting the overexpressed inflammation process.

## 1. Introduction

Both acute and chronic skin lesions heal through a process that consists of five successive phases: hemostasis, inflammation, migration, proliferation, and remodeling (maturation) [1,2]. Numerous endogenous and exogenous adverse factors can disturb the physiological healing processes, mostly during the inflammatory phase [3]. Wound tissue produces various proinflammatory cytokines and chemokines in the initial inflammatory phase, which leads to the infiltration of neutrophils [4] and macrophages to the injured sites [5]. Moderate inflammation facilitates the removal of necrotic tissue, destroys bacteria, and promotes wound healing. Conversely, excessive inflammatory infiltration interferes with normal healing processes such as collagen deposition, angiogenesis, and granulation tissue formation [6]. Therefore, the inflammation of the wound must be modulated to an adequate degree to promote wound healing while avoiding escalation that might interfere with the process.

The healing of acute wounds takes between 8 and 12 weeks [7], but sometimes, it is accompanied by uncontrolled inflammation. In such cases, proinflammatory macrophages and inflammatory mediators such as tissue necrosis factor *α* (TNF-*α*) and interleukin-1*β* (IL-1*β*) become overexpressed, and as a result, acute wounds evolve into chronic wounds [8]. In acute wounds, wound closure occurs after 8 to 12 weeks [8]. In the case of chronic wounds, due to the high degree of inflammation characterized by high levels of pro-inflammatory cytokines, healing is lengthier than in the case of acute wounds [9]. Chronic wounds remain mostly in the inflammation phase, releasing a significant amount of wound exudate [10]. Thus, chronic wounds fail to heal within 12 weeks; sometimes, it may even take them several months or even years to fully recover [9].

Until now, various biomaterials have been used to manage wound healing [11]. Any biomaterials used for that purpose are required to be well tolerated by the skin and surrounding tissue to ensure proper healing without adverse reactions. Their degradation products must be non-toxic and safely absorbed or eliminated. Also, such biomaterials should retain moisture while reducing fluid loss, thus enabling cell migration and tissue regeneration and encouraging cell adhesion, proliferation, and migration, which are essential for wound closure and tissue regeneration [12]. Promoting angiogenesis is essential to provide an adequate blood supply to the healing area. Biomaterials must have the ability to properly absorb any exudate released by wounded tissue to prevent infections and maintain proper wound hygiene.

Various studies have proven that biopolymeric nanoparticles [13] and hydrogels exhibit all those properties essential to the healing process [14]. On top of that, their healing effect can be enhanced by incorporating various bioactive substances that aid tissue regeneration, such as immunomodulators, antibiotics, and anti-inflammatory agents. The use of such biomaterials is essential in both treating and preventing infections, thus shortening the healing period, including that of postoperative wounds [15,16,17,18,19,20,21].

Anti-inflammatory drugs are capable of regulating the levels of inflammatory factors and normalizing the inflammatory response of chronic and acute wounds [22]. We selected dexketoprofen (DEX), a nonsteroidal anti-inflammatory drug (NSAID) capable of inhibiting cyclooxygenase pathways (COX1 and COX2) and widely known for its anti-inflammatory, analgesic, and antipyretic properties, to be incorporated into a thermo-sensitive hydrogel based on alginate functionalized with poly(N-vinylcaprolactam) (PNVCL). Although DEX is less commonly used for topical applications, we have considered that DEX could play a key role in modulating the inflammatory phase of wound healing, particularly in postoperative wounds, also due to its analgesic effect, which is particularly useful in postoperative pain management. That is especially important when the healing process is impaired, as in patients with diabetes, immunosuppression, venous disease, or obesity. The anti-inflammatory effects of DEX will be enhanced by alginate, a natural biopolymer known for its “ideal wound healing agent” due to its unique characteristics [21].

Alginate grafted with poly(N-vinylcaprolactam) (PNVCL) is an asset for wound healing systems due to its enhanced biocompatibility and the ability to form a gel in response to environmental conditions, such as temperature. That gelation property allows moisture retention at the wound area, promoting a moist healing environment, which is critical for faster and more efficient tissue regeneration. Additionally, the PNVCL graft improves the mechanical strength and stability of the alginate hydrogel, providing better support and protection for the wound while promoting controlled drug release, antimicrobial activity, and reduced infection risk. The role of alginates in various advanced wound dressings has been well documented [21,23,24,25,26,27]. In addition to their well-known antibacterial properties against both Gram-positive and Gram-negative bacteria, as well as antifungal activity against *Candida albicans* and antiviral effects, alginates and their derivatives have been shown to possess anti-inflammatory, regenerative, and angiogenic properties. Furthermore, they exhibit immunomodulatory and antioxidant effects, which are attributed to the release of nitric oxide (NO), reactive oxygen species (ROS), tumor necrosis factor (TNF-α), and the transcription factor NF-kB from macrophages. Those actions, along with the activation of the mitogen-activated protein kinase (MAPK) signaling pathway, contribute to their hemostatic effects, which include platelet activation and thrombin clot formation [21].

This study contributes to the advancement of alginate-based systems and their derivatives for effective wound-healing pharmaceutical formulations. Previous research has demonstrated that the addition of functional groups to the biopolymeric backbone can create non-toxic, biocompatible materials with enhanced therapeutic performance. For example, chitosan grafted with poly(N-isopropylacrylamide) has been utilized for mucosal and vaginal applications, while poly(N-vinylcaprolactam) grafted alginate hydrogels and nanoparticles are being evaluated for wound healing systems [20,21,27]. Those examples showcase the incorporation of thermosensitive moieties with a lower critical solution temperature (LCST) that is close to the temperature of the human body (around 32 °C). The key feature of those biomaterials is their ability to self-modulate based on environmental conditions such as pH, temperature, and ionic strength.

Previous studies on dexketoprofen-loaded hydrogels have primarily focused on drug delivery systems rather than wound dressing applications, highlighting a significant research gap. Lu et al. [28]. investigated a metered-dose transdermal spray for dexketoprofen, emphasizing its biopharmaceutical performance but not addressing its potential for wound healing. Similarly, Trivedi and Goyal [29] provided insights into pharmaceutical formulations of dexketoprofen, yet their study lacked specific applications in hydrogel-based wound dressings. Flores-Arriaga et al. [30] developed a PVA-based controlled-release system for a Tramadol–Dexketoprofen combination, demonstrating promising drug-release properties but focusing on systemic pain management rather than localized wound treatment. Despite the potential benefits of hydrogels in wound care, including sustained drug release, hydration, and biocompatibility, research on dexketoprofen-loaded hydrogels specifically designed for wound healing remains scarce. Given the crucial role of advanced wound dressings in accelerating healing and reducing infection risks, further studies are needed to explore the potential of dexketoprofen-based hydrogels in improving wound care outcomes.

In this context, dexketoprofen (DEX), an anti-inflammatory and analgesic drug, plays an important role by being incorporated into those systems. DEX can target the inflammatory phase of wound healing, alleviating pain and reducing inflammation, particularly in cases where the healing process is impaired (e.g., in diabetic or immunocompromised patients). When included in such biopolymer-based systems, DEX’s therapeutic effects are upgraded, improving overall wound healing outcomes. The ongoing development of functionalized alginate-based systems with tailored properties, along with the incorporation of DEX in an effort to manage inflammation, represents a promising approach to improving wound healing treatments. By fine-tuning those materials to dynamically respond to physiological conditions, we can enhance their therapeutic effectiveness and offer more efficient, personalized care for patients with acute or chronic wounds.

While much research has gone into developing advanced alginate-based systems for wound healing, this study needs to clearly highlight what makes it different. Many existing studies have looked at smart hydrogels and biopolymer modifications for drug delivery, but few have specifically focused on incorporating dexketoprofen into these systems for targeted wound treatment. What sets this work apart is its focus on creating a hydrogel that not only responds to environmental conditions but also enhances the anti-inflammatory and pain-relieving effects of dexketoprofen. By bridging the gap between smart biomaterials and personalized wound care, this study brings a fresh perspective to the field and offers new possibilities for more effective and adaptable treatments.

The current work aimed to develop and characterize a novel AA-g-PNVCL-DEX (DEXHY) hydrogel for topical application that is capable of contributing to the improvement of wound care by controlling the exacerbated inflammatory phase during the wound healing process.

## 2. Results

### 2.1. Synthesis of Dexketoprofen-Loaded Hydrogel DEXHY

**Figure 1:**
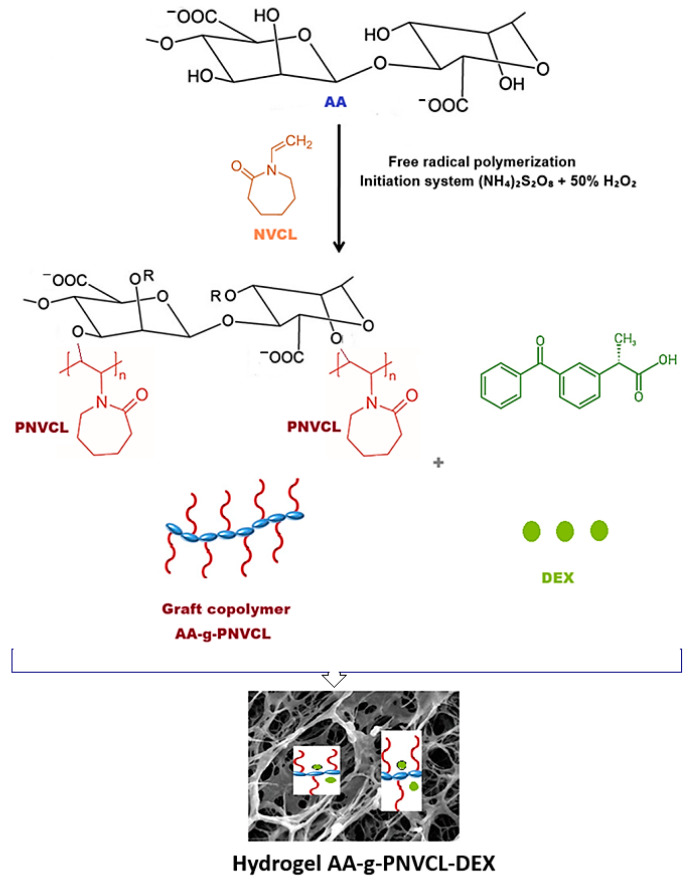
The proposed mechanism of dexketoprofen-loaded hydrogel AA-g-PNVCL-DEX synthesis.

The N-vinylcaprolactam (NVCL) monomer was chosen to be grafted onto the alginate backbone after chemically induced free radical polymerization in a nitrogen atmosphere. The initiation system represented by the mixture formed by ammonium persulfate and 50% hydrogen peroxide solution generates free radical sites on the backbone of alginate. These sites generate graft copolymers in the presence of NVCL [31]. Based on this explanation, the mechanism depicted in Figure 1 has been proposed to initiate graft copolymerization. The grafting procedure and the characterization of the synthesized copolymer were detailed and presented in previous studies [20,21].

### 2.2. Infrared Spectroscopy (FTIR) Analysis

**Figure 2:**
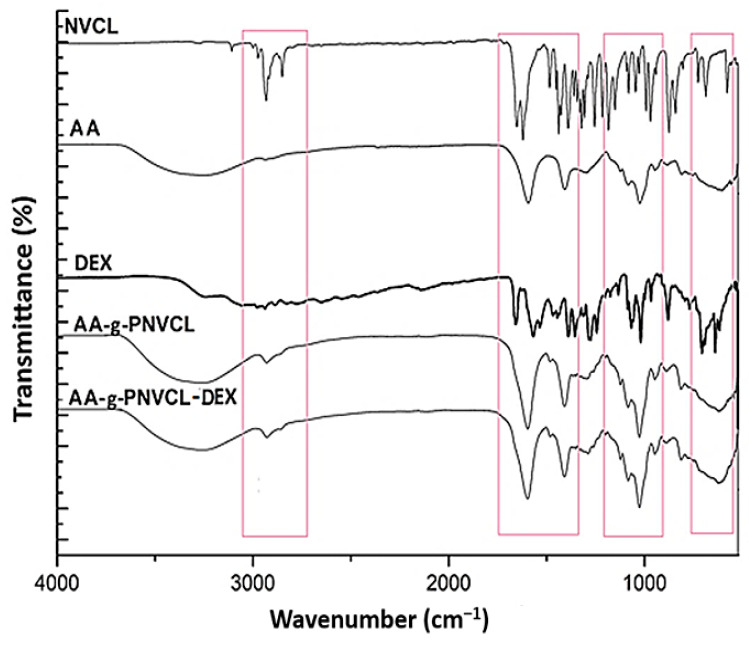
FTIR spectra of pure constituents (NVCL, AA, DEX) and the lyophilized hydrogels AA-g-PNVCL (HY) and AA-g-PNVCL-DEX (DEXHY). Red boxes represents areas where changes in absorption bands occur.

Infrared spectroscopy was used to prove the formation of polymer matrix and DEX loading. FTIR spectra of NVCL, AA, AA-g-PNVCL (HY), AA-g-PNCVL-DEX (DEXHY), and DEX are shown in Figure 2.

FTIR spectrum of NVCL (Figure 2) showed a characteristic carbonyl peak (amide I band) at 1631 cm^−1^. The peak corresponding to –C=C was observed at 1659 cm^−1^. The characteristic peaks located at 3102 cm^−1^ and 994 cm^−1^ are assigned to the vinyl group (=CH, =CH_2_). The peak observed at 1479 cm^−1^ is given by a C–N stretching vibration [32].

In the FTIR spectrum of alginate (Figure 2), a broad band centered at about 3300 cm^−1^ was determined by the stretching of hydroxyl groups, and a low-intensity band at about 2850 cm^−1^ attributed to –C–H stretching vibration was observed. The peak found at 1795 cm^−1^ was attributed to the –C=O stretching vibrations from the –COCH_3_ group. The peak at 1180 cm^−1^ was assigned to –CH–OH groups [33]. The peak at 1017 cm^−1^ indicates the –CH–O–CH– stretching.

The FTIR spectrum of pure dexketoprofen substance (Figure 2) shows a characteristic absorption band at 1020 cm^−1^. The band observed at 771 cm^−1^ corresponds to the stretching vibration of the =C–H bond. An intense and sharp absorption band appears at 1659 cm^−1^ (corresponding to the C=O bond) and 1569 cm^−1^ (corresponding to the NH_2_ group). The band observed at 3255 cm^−1^ is attributed to the stretching of the O–H bond, while the absorption band located at 3057 cm^−1^ could be attributed to the stretching of the C–H group by the aromatic proton [34]. The absorption band appearing at 2979 cm^−1^ is due to the stretching of the C–H bond from CH_3_ groups.

In the IR spectra of HY (Figure 2), we observed the disappearance of the broad band between 3000–2800 cm^−1^ attributed to NCVL and an intensification of the broad band in the range of 3000–3500 cm^−1^ corresponding to alginate. The shift of the characteristic C=O peak from 1744 cm^−1^ to 1795 cm^−1^ is suggested to be due to the destruction of H-bonds between AA carboxyl groups, which corresponds to the involvement of carboxyl groups in H-bonding.

In the DEXHY spectrum (Figure 2), the peak at 1430 cm^−1^ attributed to C-N stretching is intensified. An intensification of the bands at 1039 cm^−1^ and 1089 cm^−1^ was also found. This demonstrates the creation of hydrogen bonds between HY and DEX [20]. The band observed between 3000 and 2933 cm^−1^ suggests the elongation of the aliphatic C–H bonds. The intensification of the N–H and O–H stretching vibrations was noticed at 3332 cm^−1^ and 3447 cm^−1^, respectively. These changes suggest that the polymer system was formed and dexketoprofen was encapsulated in the polymer matrix.

### 2.3. Differential Scanning Calorimetry (DSC) Analysis

**Figure 3:**
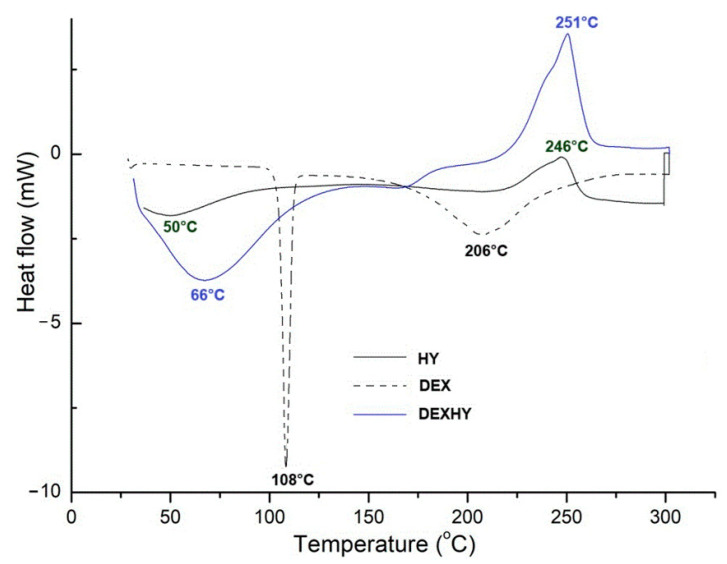
DSC thermograms of the pure drug (DEX), unloaded hydrogel (HY), and dexketoprofen-loaded hydrogel (DEXHY).

The DSC measurements of the pure drug, the hydrogel, and the dexketoprofen-loaded hydrogel were performed, and the thermograms of the three compounds are represented in Figure 3.

The DSC thermogram of the drug confirms the purity of dexketoprofen trometamol. It illustrates a sharp endothermic peak at 108 °C and a broad endothermic peak at 206 °C (Figure 3). On the DSC curve of lyophilized HY, a first transition at 50 °C and a broad exotherm at 246 °C were observed. The DSC thermogram of DEXHY hydrogel (Figure 3) shows a major endothermic peak at 66 °C and a broad exothermic peak at 251 °C. The endothermic peak of HY from 50 °C shifted to 66 °C and the exothermic peak from 246 °C shifted to 251 °C in DEXHY.

### 2.4. Scanning Electron Microscopy (SEM) Analysis

**Figure 4:**
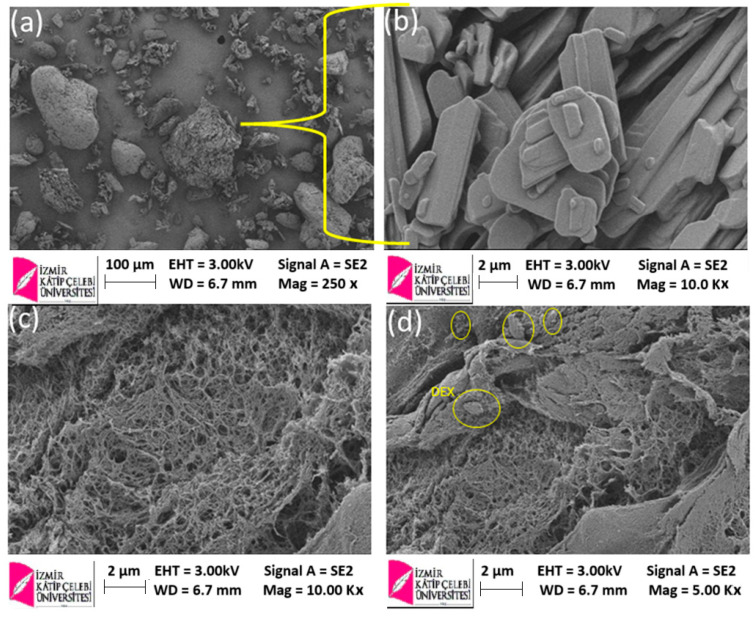
SEM images for pure dexketoprofen (DEX) at different magnifications: 250× (**a**); 10.00 K× (**b**); lyophilized hydrogel HY recorded at a magnification of 10.00 K× (**c**); lyophilized hydrogel DEXHY recorded at a magnification of 5.00 K× (**d**).

The obtained hydrogels were lyophilized and analyzed morphologically by scanning electron microscopy (Figure 4). As observed in Figure 4c, the lyophilized copolymeric matrix (HY) showed a porous structure with different pore sizes and shapes but uniformly distributed throughout the polymeric matrix. The formed pores will influence the absorption of physiological solutions, the swelling of water, and the diffusion of solute (DEX) into and out of the gel network. Under the electron microscope, dexketoprofen particles looked predominantly like thin and elongated platelet-shaped particles several microns long, on average, as observed in Figure 4a,b. The SEM image of DEXHY (Figure 4d) confirmed the incorporation of the drug inside the network, as the presence of DEX reduced the porous appearance of the material, giving it a rough appearance. The distribution of the drug particles was uniform, showing a filling effect of the porous polymer morphology. The HY has pores measuring between 12 µm and 86 µm (Table 1), while in DEXHY, the pore size ranges from 10 µm to 72 µm. We consider that the porous structure of the hydrogel network strongly influences the swelling capacity, drug loading, and release capacity.

### 2.5. Swelling Behavior of Unloaded Hydrogel and Dexketoprofen-Loaded Hydrogel

**Figure 5:**
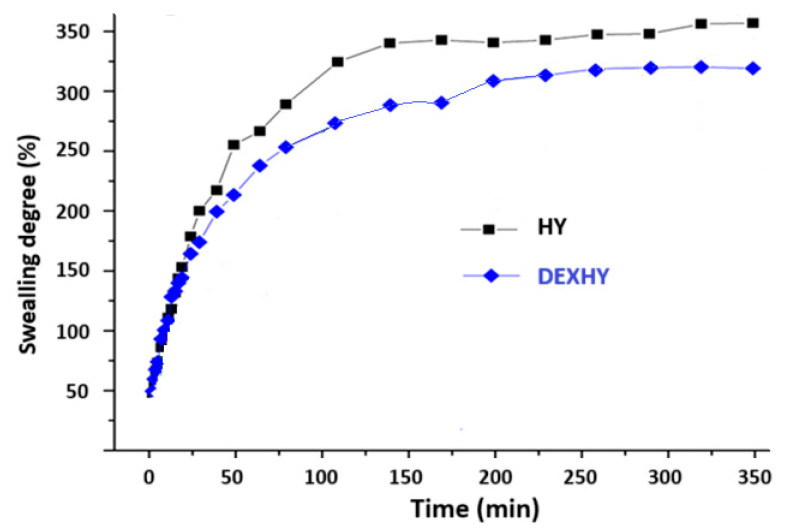
Swelling degree of lyophilized hydrogel HY and loaded hydrogel DEXHY in phosphate buffer pH = 7.4 at 37 °C. The data are expressed as mean ± standard deviation (SD) with n = 3.

The swelling behavior of the lyophilized matrices, both with and without the drug, was analyzed in PBS at pH 7.4 and 37 °C, as shown in Figure 5. The determined maximum swelling degree was 350% for the unloaded matrix (HY) and 300% for the DEX-loaded matrix, with both matrices reaching a plateau after approximately 3 h. As shown in Figure 5, both matrices exhibited rapid swelling, reaching over 80% of their total swelling capacity within the first hour, indicating a fast swelling rate typical of a porous structure. The reduced swelling ability of the DEX-loaded matrix was consistent with the SEM images, which revealed a more compact structure with smaller pores. This suggests that dexketoprofen may be acting as a filler, being entrapped within the polymeric matrix through electrostatic interactions.

The swelling kinetic parameters of HY and DEXHY hydrogels evaluated using the Fick diffusion model are given in Table 2.

### 2.6. Drug-Loading Capacity and Release

**Figure 6:**
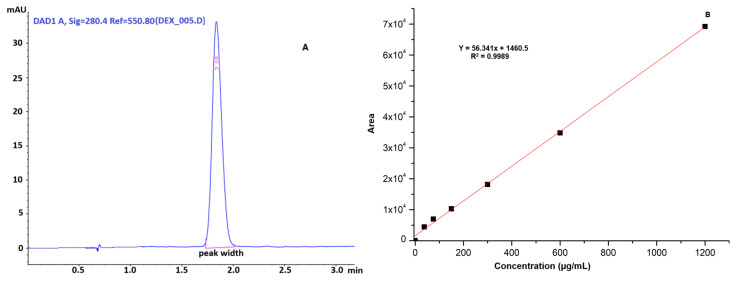
HPLC chromatogram of the loaded hydrogel DEXHY (**A**) and the calibration curve for drug release (**B**) (red colored line represents the peak width).

The quantification of dexketoprofen loaded into the hydrogel was calculated based on the developed HPLC method. The HPLC chromatogram of the dexketoprofen-loaded hydrogel and the calibration curve for the evaluation of DEX release from DEXHY are shown in Figure 6.

**Figure 7:**
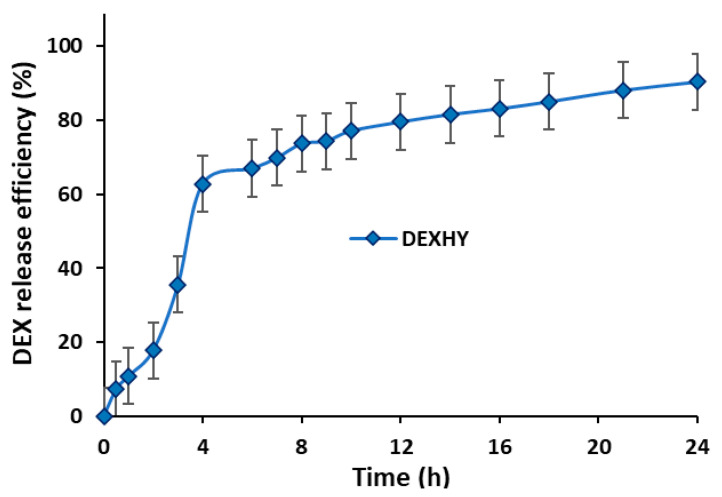
DEX release efficiency from DEXHY hydrogel. Data are mean ± SD within ±2 (n = 3).

The retention time of dexketoprofen was determined to be 2.18 min. The HPLC method used for DEX quantification was validated in accordance with established guidelines. Linearity was assessed using six different concentrations ranging from 37.5 µg/mL to 1200 µg/mL, yielding the equation y = 56.341x + 1460.5 with an r^2^ value of 0.9989. The method demonstrated high precision, with values of 99.603 ± 0.413%, 100.125 ± 0.106%, and 99.675 ± 0.282% (mean ± SD) for concentrations of 75, 150, and 300 µg/mL, respectively (n = 6). Method recovery was satisfactory, with an RSD value below 2%. The limit of detection (LOD) and limit of quantification (LOQ) were determined to be 0.127 µg/mL and 0.385 µg/mL, respectively. The developed method was confirmed to be accurate, as indicated by RSD values below 2% for both repeatability and intermediate precision at concentrations of 75, 150, and 300 µg/mL. Analysis of the data revealed that DEXHY encapsulated 66.40% of the initially loaded DEX (0.5% w/w relative to the polymer matrix). The concentration of released DEX was calculated according to the standard curve previously plotted in Figure 6. The in vitro DEX release profile from the lyophilized matrix is represented in Figure 7.

The data indicated a rapid release phase occurring within the first 4 h, followed by a slower, sustained release up to 24 h. The release efficiency of DEX from the DEXHY sample was 98.25%. Various kinetic models were applied to analyze the in vitro release profile of DEX from DEXHY. The estimated release kinetic parameters, derived from mathematical models for the freeze-dried DEXHY hydrogel, are summarized in Table 3.

The results suggest that the transport/release mechanism is predominantly controlled by diffusion.

### 2.7. Bioadhesion Properties

**Figure 8:**
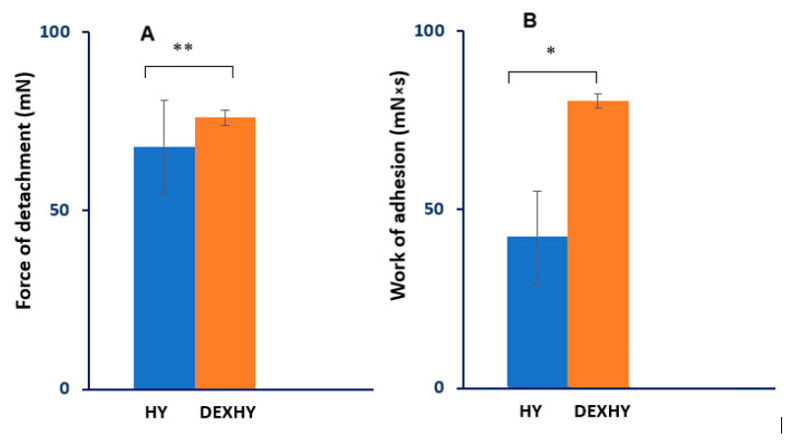
Bioadhesion properties of hydrogels HY and DEXHY, determined as the detachment force (**A**) and work of adhesion (**B**) (* *p* < 0.05, ** *p* < 0.01).

The bioadhesivity of both unloaded and dexketoprofen-loaded hydrogels was studied. The detachment force of the sample from the dialysis membrane surface and the mechanical work of adhesion are given in Figure 8. Each value represents the mean ± mean standard error from three independent experiments (n = 3).

### 2.8. Hydrogel Cytocompatibility

**Figure 9:**
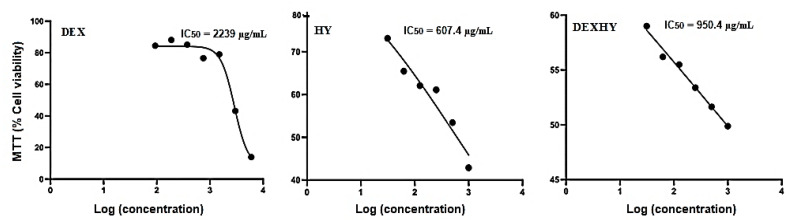
IC50 values for DEX, HY, and DEXHY determined by the cell viability assay.

To determine the cytotoxicity and IC50 values (IC50 is the dose of the active substance required to inhibit 50% cell proliferation) of the pure active substance and the hydrogels, the MTT assay was performed. Figure 9 describes the cytotoxicity results and calculated IC50 values for all samples studied—DEXHY, HY, and DEX (IC50 values were determined by applying the sigmoidal mathematical model of the GraphPad program, (version 10). The IC50 values found for the samples we tested were 2239 µg/mL for DEX, 607.4 µg/mL for HY, and 950.4 µg/mL for DEXHY.

**Figure 10:**
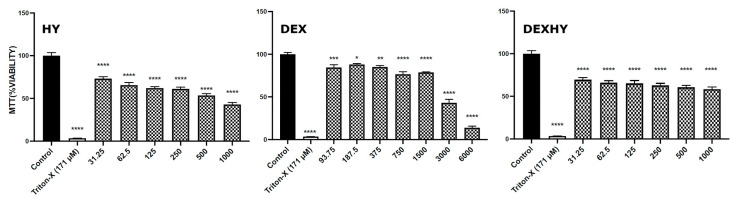
MTT cell viability of 3T3-L1 cells for DEX solutions, HY, and DEXHY (n = 3; * *p* < 0.05, ** *p* < 0.01, *** *p* < 0.001, **** *p* < 0.0001 vs. control, error bars = SD).

Figure 10 presents the cell viability (%) measured by the MTT assay, which reflects mitochondrial dehydrogenase activity following cell exposure to HY, DEX, and DEXHY. Each result represents the mean viability ± standard deviation (SD) of three parallel experiments (n = 3).

### 2.9. In Vitro Wound Healing Assay

**Figure 11:**
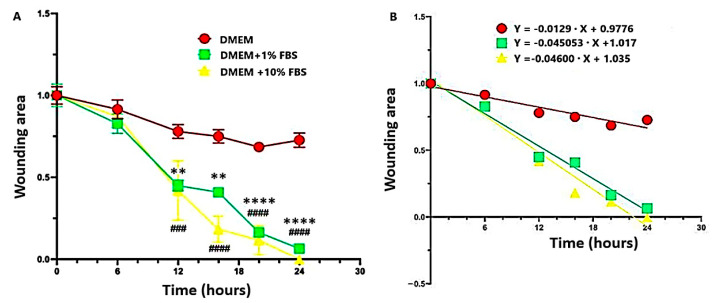
Comparison of scratch closure rates (**A**) and the slopes of the scratch closure rates (**B**) for control medium DMEM, DMEM +1% FBS, and DMEM + 10% FBS as a function of time rates (** *p* < 0.01, **** *p* < 0.0001 vs. control; ^###^ *p* < 0.001 and ^####^ *p* < 0.0001 vs. control).

The scratch test method was used to evaluate the in vitro wound closure effects of DEX, HY, and DEXHY hydrogels. Figure 11 shows the time-dependent rates of scratch closure on 3T3-L1 mouse fibroblast cultures in the presence of DMEM, DMEM + 1% FBS, and DMEM + 10% FBS as control media and as a function of the slopes obtained in the case of each incubation medium. That figure, within a scale from 0 to 1, showed the decreasing wound area in the case of the application of various mediums, and the optimized medium was established for which the closure percent is maximum.

**Figure 12:**
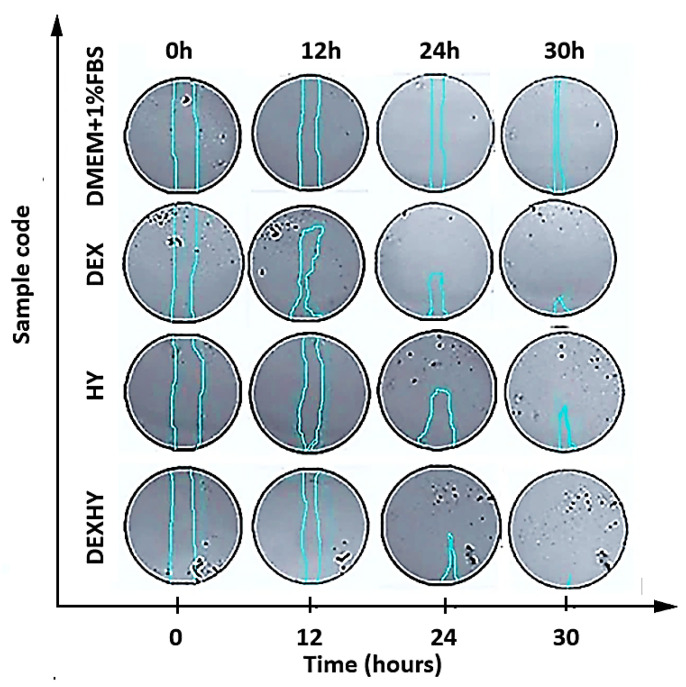
The images of the scratches in each well and their closure according to the function of time at 0 h, 12 h, 24 h, and 30 h.

Figure 12 represents the microscopic images of the scratch on cell cultures for each sample tested over the 30 h duration of the experiment. The wound edges are marked by the blue lines.

Closure rates were quantified by ImageJ software v1.54 but can also be visually observed, especially when comparing the beginning of the experiment (0 h) with its end (30 h).

**Figure 13:**
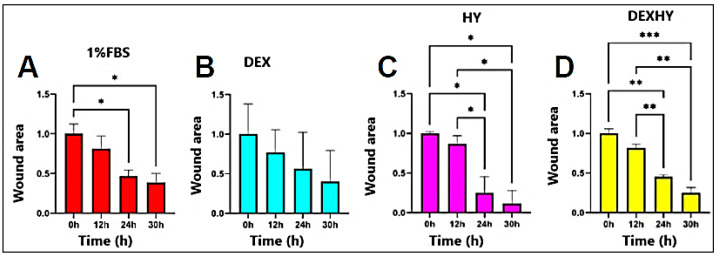
Wound areas of 3T3-L1 cells incubated with control (DMEM + 1% FBS), DEX, HY, or DEXHY until the 30th h. (**A**) Control, (**B**) DEX, (**C**) HY, and (**D**) DEXHY groups (*p* < 0.05 *, *p* < 0.01 **, *p* < 0.001 ***).

Figure 13 presented the closure of the simulated wound when the optimal medium and the tested samples were applied within the studied time interval. The closure activity was compared between the tested samples and images of scratch areas from the time points 0, 12, 24, and 30 h. It was observed that the activities of DEX and HY solutions were much higher than the DEXHY solution after 12 h; however, at 24 h and 30 h, a better activity was determined for DEX and DEXHY solutions.

## 3. Discussion

Various formulations of DEX administration have been developed, but little research is reported for its cutaneous or topical administration [35,36]; moreover, none of them include the use of alginate. Several considerations were taken into account when choosing the DEXHY formulation ingredients. Primarily, all components have to be non-toxic in order to be used in a drug release system [37]. Alginate is a biomaterial that can be easily modified by chemical and physical reactions for its use in biomedicine, including wound healing [38], drug delivery [39], and tissue engineering applications [40]. Poly(N-vinylcaprolactam) is the most popular temperature-sensitive polymer that exhibits a lower critical solution temperature (LCST) between 25 and 50 °C. For this reason, it is very attractive for biomedical applications [41]. In our study, sodium alginate was functionalized with PNVCL by grafting techniques, obtaining an improved polymer matrix able to respond to the natural temperature variations of the human body, which improves the ability to deliver the active substance to the site of action. On the other hand, the loading of the copolymeric matrix was carried out with dexketoprofen, a nonsteroidal anti-inflammatory, to decisively intervene in the inflammatory stage of the wound healing process, which can be disturbed in some diseases (diabetes or venous diseases). This can avoid the transformation of an acute wound into a chronic wound since both acute and chronic wounds are associated with inflammation; moreover, patients will also benefit from the analgesic and antipyretic action of dexketoprofen, particularly useful in post-operative/wound care.

The intensification of the broad band of AA in the region of 3000–3500 cm^−1^ and the disappearance of the broad band of NVCL (3000–2800 cm^−1^) in the FTIR spectra of HY suggest the possible chemical interaction and formation of new bonding between AA and NVCL (Figure 2). The peak recorded at 1621 cm^−1^ was attributed to the carbonyl groups in the amide (N, N′-dialkyl amide) ring structure of the graft copolymer (AA-g-PNVCL) [42]. After the syntheses of DEXHY, it was observed that some FTIR characteristic dexketoprofen peaks at 1659 cm^−1^, 1571 cm^−1^, 1536 cm^−1^, 1020 cm^−1^, 881 cm^−1^, 771 cm^−1^, and 641 cm^−1^ [43] disappear, suggesting the occurrence of drug entrapment in the polymeric structure (Figure 2). When comparing the spectra of HY and DEXHY with that of DEX alone, it can be observed that there are changes in the intensity and shape of signals in the range of 3600–2800 cm^−1^ and at 1651 cm^−1^.

From the thermogram of the pure drug (DEX), a sharp endothermic peak at 108 °C was determined, which is consistent with other reports from the literature. Bruni et al. [44] analyzed in detail the thermal behavior of dexketoprofen by differentiating the two polymorph forms of the drug. Similarly, they determined a melting peak at 106 °C corresponding to a predominant form A within a mixture. Another thermal event determined at 206 °C was attributed to the beginning of the degradation of the drug. The incorporation of the drug within the polymeric matrix led to the decrease in intensity of the peak from 108 °C and even to its disappearance, as also reported in other studies. The incorporation of DEX indicated that a homogenous matrix was formed with a dominant amorphous structure, and thus, a loss of drug crystallinity occurred [43]. As observed in Figure 3, from the thermogram of the drug-loaded hydrogel compared with the simple lyophilized biopolymeric matrix (HY), a small peak around 170 °C was assigned to the presence of the drug within the polymeric matrix. The transition at 50 °C and the shift to 66 °C in the case of the drug-loaded matrix were attributed to the moisture evaporation from the polymeric matrix, and the broad exotherm peak at 246 °C marks the beginning of the degradation of the polysaccharide chains. Similarly, Lozinsky et al. [45] studied the degradation behavior of biopolymeric matrices containing N-vinyl caprolactam moieties and reported a significant decrease in the molecular weight of the polymer, starting with a temperature of 290 °C in the form of depolymerization. Additionally, the thermal degradation of alginate-based matrices is also supposed to start around 200 °C, which is consistent with other reports [46].

In SEM analysis, a similar appearance of DEX was also found by Román et al. [47] and Bruni et al. [44], confirming a predominant polymorphic structure A of the drug. The porous structure of the polymeric matrix allowed the drug to be incorporated inside the matrix, resulting in DEX-loaded hydrogels. The presence of the drug inside the polymeric matrix was proved not only by the drug-loading capacity tests but also by the SEM images, in which stick-like particles were identified throughout the hydrogel structure (Figure 4d). Additionally, the presence of DEX seemed to reduce the porous appearance of the hydrogel and had a tough filing effect. It was determined that the HY has pores ranging between 12 µm and 86 µm (values are summarized within Table 1), and the DEXHY’s pore sizes ranged between 10 µm and 72 µm. The reduction in pore sizes was further confirmed by the swelling behavior of the hydrogels both with and without the drug. The presence of the drug within the matrix resulted in a more compact morphological structure, which exhibited a lower swelling capacity. Hydrogels with interconnected pores are useful for cell growth because they provide a large surface area for cell adhesion [48] and migration [49].

Swelling behavior is an important step in the characterization of hydrogels, as it directly influences the mechanism and kinetics of the release of loaded drugs. Also, if considering their use in wound healing, it is important to know the swelling behavior since the hydrogel must absorb the fluids resulting from the wound. Since the pH of the wound microenvironment is 7.4 [50] and the body’s cells and enzymes function best at 37 °C [51], swelling studies were performed in PBS with a pH of 7.4 at 37 °C, which simulates physiological conditions. According to the swelling behavior (Figure 5) and SEM images (Figure 4), the obtained polymer systems can be classified as porous hydrogels. The first impact of the hydrogels with the buffer solution produced rapid swelling due to the pores having a higher absorption capacity than simple absorption by diffusion. The degree of swelling of the hydrogels reached a maximum after 50–55 min, followed by a steady state. In many research articles for Fickian diffusion, n is reported close to 0.5 or over 0.5. In our study, the diffusion exponent n recorded values lower than 0.5, which suggests that the solvent penetration speed is lower than the relaxation speed of the polymer chains. This behavior, which is a subclass of Fickian diffusion, corresponds to a diffusion called “less Fickian”. The fact that the hydrogels exhibited a rapid swelling process with an increased degree of swelling confirms their possibility to be used as flexible biomaterials with an increased degree of hydration in tissue engineering applications [52]. The copolymer hydrogels showed high values of the degree of swelling, also due to the presence of an even greater amount of hydrophilic functional groups originating from the components [53].

The ability of the DEXHY formulations to release DEX was assessed using Franz diffusion cells. According to Figure 7, the release process of DEX from DEXHY manifests a burst effect in the first 4 h, releasing around 65% of the total amount released. After this period, release occurs slowly and in very small amounts. The interaction between the copolymer and DEX may cause significant changes in the release behavior of the drug. The initial burst release of dexketoprofen can be attributed to the drug trapped on the surface of the hydrogel during the preparation process. The large porous surface area of the hydrogel allows the deposition of the drug through molecular adsorption on the surface. The deprotonation of the –COOH functional groups at pH = 7.4, both for AA and DEX, can produce strong electrostatic repulsion forces that lead to an increase in the degree of swelling of the hydrogel and, therefore, to an increase in the drug release percentage. Burst release may be optimal in the first stage of wound healing for immediate relief [36]. The sustained release of DEX after 5 h until 24 h can be explained by the fact that the released drug was adsorbed in the HY matrix due to its porosity. The kinetic release models can be used to predict the distribution of drugs inside a polymer matrix and depend on the outcome of drug loading in the given carrier [54]. The release data were fitted to different kinetic models such as zero order, first order, Higuchi model, and Korsmeyer–Peppas. Table 3 shows clearly that the selection criteria of the Korsmeyer–Peppas kinetics were more likely met than the criteria of the other kinetic model versions (r^2^ = 0.9883). The results presented in Table 3 suggest the occurrence of Fickian diffusion mechanisms (n = 0.5336). This implies that drug release occurs at a rate equal to the relaxation rate of the polymer chains. For Fickian diffusion, n values close to 0.5 or >0.5 have been reported in most published articles.

The hydrogels used in the treatment of wounds must have good bioadhesiveness, which is crucial for ensuring sustained stability. This influences hemostasis and maintains optimal moisture levels in the wound [55]. Bioadhesion represents the process by which natural and synthetic materials adhere to biological surfaces [56]. Polymers are often used in pharmaceutical formulations as bioadhesive excipients for their adhesion to biological membranes when prolonged contact with the skin is desired. Different in vitro and in vivo methods were applied to determine the degree of bioadherence of hydrogels. In vitro methods are preferred because they are cost-effective, easy to perform, and less time-consuming [57]. Due to its safety, effectiveness, and non-immunogenesis, alginate is the most used bioadhesive polymer for drug administration [58]. The tested samples show good bioadhesion to the substrate due to alginates that contain many hydrophilic (–OH) and carboxyl (–COOH) groups that stimulate the hydrogel wettability. The –OH group is found to have the highest adhesion strength to cells, followed by –COOH. The hydrogel wettability is considered the first mechanism of adhesion. The wetting and swelling of the scaffolds, the interpenetration of polymer chains, and finally, the chemical bonds are important steps in controlling bioadhesion. The unloaded hydrogel showed lower values of detachment force and work of adhesion (Figure 8), possibly due to PNVCL, which has a low bioadhesive strength due to its hydrophobic chain (carbon–carbon) and hydrophilic cyclic amide side groups. An increase of 49.3% in bioadhesion capacity was found when DEX was encapsulated in the hydrogel (Figure 8) due to the increased number of functional groups that can adhere to the substrate.

The MTT assay on mouse fibroblasts (3T3-L1) was used to determine the cytotoxicity and IC50 values of the pure active substances and formulations. The MTT assay is used to investigate biological processes and assess cell viability by comparing experimental cultures with control ones [59]. Cytotoxicity data on dexketoprofen are limited. However, in a study by Gökçe et al., the IC50 value of DEX was found to be 683 µM [60]. The effect of the drug was studied on erythrocyte hCA I and hCA II. The IC50 value of the DEXHY hydrogel was relatively higher than that of HY unloaded hydrogel (Figure 9). Loading the matrix with DEX brought a benefit in terms of decreased toxicity and enhanced wound healing capacity. These data confirm our choice regarding the polymer matrix and the medicinal substance.

The value of cell viability (%) after the exposure of cells to DEX was 89.49% for the DEX solution at 187.5 µg/mL and 87.04% for the DEX solution at 375 µg/mL (Figure 10). A decrease in the cell viability values (%) was found after the exposure of the cells to the HY hydrogel, this being 71.06% for the 31.25 µg/mL HY solution, and it is noted that the values decreased little by little with the increase in concentration (Figure 10). Values for cell viability that were comparable with those of the HY hydrogel were also recorded (%) after exposure of cells to the DEXHY hydrogel, and they were around 75.4% for all concentrations of DEXHY solutions tested (Figure 10). DEXHY showed low and lower cytotoxicity than the unloaded hydrogel (HY). This was possible due to the incorporation of dexketoprofen, which has very low cytotoxicity and a high cell viability value. Experiments have shown that if the cell viability of a material is greater than 70% compared to the control sample, then the material is non-cytotoxic [61]. The prepared hydrogel DEXHY was found to be promising for clinical use. No cytotoxic effect was shown for the prepared hydrogels at different concentrations because the cell viability was found to be greater than 94% after incubation for 24 h, as indicated in Figure 10. Therefore, we can conclude that the developed DEXHY hydrogel could be used for medical applications in the near future.

Even though studies on the direct effects of NSAIDs on wound healing are scarce, some of them claim that they can have a beneficial effect by controlling inflammation [62]. Certain hydrogels loaded with anti-inflammatory drug active substances, such as diclofenac sodium [63], aceclofenac [64], ketoprofen [65], piroxicam [66], dexamethasone [67], and ibuprofen [68], have been reported in the literature. The in vitro scratch wound model was used for examining cell migration and wound closure in the presence of DEX, HY, and DEXHY solutions. Wound closure was monitored at 0, 12, 24, and 30 h intervals, and the relative wound area (%) was determined. After 12 h, the processes of wound closure started for all samples (Figure 12). It was observed that there was a significant difference between the closing rates of DMEM and DMEM supplemented with 1% or 10% FBS after 12 h (Figure 11), which can also be determined quantitatively from the slopes of each curve (Figure 11). Also, a minimal distinction was noted between 1% FBS and 10% FBS in terms of scratch closure activity. Therefore, we decided to continue the experiments with the control medium DMEM + 1% FBS. The unloaded hydrogel, HY, showed a high scratch-closing effect compared to the control medium (DMEM + 1% FBS) at 30 h, which supports our choice of the AA-g-PNVCL copolymer matrix. The scratch-closing effect of DEX was different from the control medium at 30 h but also at earlier time points (Figure 12). The dexketoprofen-loaded hydrogel caused a higher rate of closure than both the control medium and HY alone, demonstrating the ability of DEXHY to support cell proliferation (Figure 13). All these results corroborated with the data from the cytotoxicity studies and demonstrated our working hypothesis—that our DEXHY hydrogel can stimulate cell proliferation and migration and induce faster wound closure. This in vitro wound healing potential strongly suggests that the dexketoprofen-loaded hydrogel formulation may exhibit effective wound healing activity in vivo as well, with a premise for further preclinical and clinical studies. A similar result was mentioned in a study carried out by Büyükfidan et al. [69], which demonstrated that epithelialization, contraction, and angiogenesis were more advanced when dexketoprofen was used, even if dexketoprofen caused disruption in collagen organization. Our results are discordant with the results of previous studies investigating the effects of DEX and many other NSAIDs on wound healing [70]. This difference could be explained by the use of other cell lines. The effects of dexketoprofen on the viability and migration of mouse fibroblasts were also investigated. Fibroblasts play an important role in wound healing and contribute to proliferating, migrating, and synthesizing collagen [71,72].

## 4. Materials and Methods

### 4.1. Materials

Sodium alginate (AA), CAS 9005-38-3 with 15 and 25 cP viscosity for the 1% solutions, N-vinylcaprolactam (NVCL), CAS 2235-00-9, and the initiator system represented by ammonium persulfate (APS)—hydrogen peroxide—50% solution were Sigma-Aldrich products (St. Louis, MI, USA). The copolymer AA-g-PNVCL was purified using a dialysis bag with a cutoff of 3.5 kDa obtained from Scienova GmbH (Jena, Germany). Dexketoprofen trometamol (DEX) was purchased from Sigma Aldrich, Saint Louis, MO, USA.

Reagents used in cytotoxicity and wound healing tests included the following: Dulbecco’s Modified Eagle’s Medium (DMEM); fetal bovine serum (Sigma-Aldrich, Hamburg, Germany); penicillin–streptomycin solution (Sigma-Aldrich, Hamburg, Germany); 171 µM Triton-X solution (Positive Control) (obtained from Sigma-Aldrich, Hamburg, Germany); sterile PBS (obtained from Thermo Scientific, Lengnau, Switzerland); ethanol 70%; and 3-(4,5-dimethyl-2-thiazolyl)-2,5-diphenyl-2H-tetrazolium bromide (MTT) obtained from Sigma-Aldrich, Hamburg, Germany. All reagents were used as purchased and not subjected to any preprocessing.

### 4.2. Obtaining the Dexketoprofen-Loaded Hydrogel DEXHY

#### 4.2.1. Synthesis of Alginate Copolymer Grafted with Poly(N-Vinylcaprolactam)

Poly(N-vinylcaprolactam) grafted alginate copolymer (the polymer matrix) was obtained by mixing a 2% (*w*/*w*) sodium alginate aqueous solution with a solution of NVCL dissolved in a small amount of dimethylformamide (DMF). The polymerization reaction of NVCL was carried out for 5h in the presence of ammonium persulphate and 50% hydrogen peroxide solution as an initiating system under nitrogen flow within a ratio of 0.1% against NVCL, with continuous stirring at 75 °C. The separation of the copolymer from the unbound free monomer was achieved by precipitating the resulting solution in cold ethanol. To remove unreacted reagents, the obtained product was purified for 7 days using a dialysis membrane (3.5 kDa cutoff) against double-distilled water. The dialysis medium was changed twice daily to maintain the difference in ion concentration. The final purified product was lyophilized. The percentage and efficiency of grafting were calculated using the procedure reported by Ziminska et al. [73].

#### 4.2.2. Preparation of Unloaded Hydrogel AA-g-PNVCL (HY)

The unloaded hydrogel (HY) was prepared following the procedure below: the copolymer was dissolved in twice-distilled water (0.1%, *w*/*w*), and the resulting solution was precipitated in ethanol; the obtained hydrogel was separated and subsequently lyophilized, finally obtaining the unloaded hydrogel, HY.

#### 4.2.3. Preparation of Dexketoprofen-Loaded Hydrogel AA-g-PNVCL-DEX (DEXHY)

For the preparation of the hydrogel loaded with dexketoprofen (DEXHY), an amount of polymer matrix was dissolved in water within a concentration of 0.1 wt.%. Then, 0.5% (*w*/*w*) of DEX solution was added to the polymeric solution (Figure 1). The theoretical loading amount of DEX in the matrix was established against the amount of polymer used. The mixture was stirred for 6 h at room temperature and 300 rpm to stimulate the formation of bonds of DEX with the hydrophilic groups of the biopolymer. The resulting solution was precipitated in ethanol, and the formed gel was separated and lyophilized, finally obtaining the dexketoprofen-loaded hydrogel, DEXHY. Lyophilized hydrogels were deposited in plastic bags and stored at 20 °C. In those conditions, the hydrogels showed no macroscopic effects of dissolution after 6 weeks of storage.

The description and codes of the hydrogels that were prepared, analyzed, and characterized are summarized in Table 4.

### 4.3. Structural Analysis by FTIR

Attenuated total reflectance Fourier transform infrared (ATR-FTIR) spectra of DEX, HY, and DEXHY lyophilized hydrogels were recorded using an ATR-FTIR instrument (Shelton, CT, USA). A quantity of dry hydrogel was ground to its powder form and placed on the ATR crystal. Spectra were recorded at room temperature in the range of 4000–500 cm^−1^_._

### 4.4. Analysis by DSC

Differential scanning calorimetry of the DEX and hydrogels was performed using a Perkin Elmer Diamond DSC instrument (Grovewood Rd, Misterton, UK) under an inert nitrogen atmosphere with a gas flow rate of 50 mL/min. The samples of approximately 10 mg DEX and dry hydrogel were added to an aluminum crucible and subjected to an initial isotherm at 30 °C for 5 min, then at a heating rate of 10 °C/min, in the temperature range of 30–300 °C.

### 4.5. Morphological Analysis by SEM

Hydrogel cross-sections were metalized with a thin layer of gold using a spray coater in an inert environment using a vacuum evaporator. The obtained samples were analyzed using scanning electron microscopy (SEM, Hitachi, Japan). The porosity of hydrogels was determined by measuring the pore size with ImageJ software v1.54. Three replicate samples were subjected to SEM analysis.

### 4.6. Swelling Study of Hydrogels

A volumetric method using a QIAquickRSpin Column 50 device (Miami, FL, USA) provided with a cellulose membrane interconnected to a microsyringe was applied for the determination of the degree of swelling. The cellulosic membrane prewetted with phosphate buffer (pH = 7.4) does not allow the passage of the sample. Before swelling measurements, disk specimens were cut from the prepared hydrogels. Hydrogel samples were precisely weighed on the analytical balance (m = 10 mg) and were inserted into the QIAquickRSpin Column 50 device. To determine the swelling capacity of the hydrogels, 1 mL of PBS solution pH = 7.4 was added to the microsyringe. The volume absorbed by the samples kept in the oven at a temperature of 37 °C was determined for 350 min. Three parallel determinations were performed for each sample, and the expressed results are the mean values.

The degree of swelling (DS) % of hydrogels was calculated using the following Formula (1):

DS% = (W_h_ − W_d_)/W_d_ × 100, (1)

where W_h_ = W_d_ + V_abs(t)_.

W_h_ represent the mass of solution absorbed at time t, and W_d_ represents the mass of the sample. Kinetic swelling parameters n and k (min^−1^) were determined in PBS pH = 7.4 for HY and DEXHY hydrogels at 37 °C.

### 4.7. Drug-Loading Efficiency and Release Kinetics of Dexketoprofen

For the quantification of dexketoprofen loaded in DEXHY, an HPLC method was applied using a Shimadzu Nexera LC-40-XR system (Shimadzu, Kyoto, Japan) fitted with a UV-Vis Series SPD-40V detector (Shimadzu, Kyoto, Japan). A C18 column (2.1 × 150 mm, Waters CORTECS 2.7 μm) and a mobile phase that contains water/formic acid—99.9/0.1 (*v*/*v*) and acetonitrile were used. The injected sample was 10 μL, the run time was 10 min (0.8 mL/min), and the optimal mobile phase ratio was A (60%):B (40%). DEX detection was performed in UV-Vis at 254 nm. The quantitative analysis of DEX was performed based on retention times and peak areas, respectively. LabSolutionDB Software (Version 6.106SP1) was used for peak integration.

For the calibration curve, a stock solution (containing 2000 µg/mL) of DEX standard substance was prepared by dissolving in distilled water. From the stock solution, serial dilutions (37.5, 75, 150, 300, 600, and 1200 µg/mL DEX) were prepared. Experiments were performed in triplicate. The calibration curve was obtained by plotting the mean area of the three determinations ± SD as a function of DEX concentration. The slope and intercept were determined from AU, and using Equation (2), DEX was quantified from DEXHY.

AU = Slope × Concentration + Intercept(2)

The release study was performed using a phosphate buffer solution (pH = 7.4) as the medium for 24 h. An amount of 0.3 g of DEXHY was introduced into a volume of 50 mL of phosphate buffer (pH = 7.4) using a dialysis membrane. The mixture was then stirred at a rotation speed of 80 rpm and a temperature of 37 °C. At different time intervals, 500 μL of sample was collected. The collected volume was filtered through a 0.22 μm Watman filter, and then 10 μL of sample was injected using the conditions of the HPLC method. The experiment was performed in triplicate.

The concentration of DEX (%) released from DEXHY in simulated fluid was calculated using the following Equation (3):

DEX released (%) = C_1_/C_0_ × 100% (3)

where

C_1_ = concentration of DEX released from DEXHY in simulated fluid;

C_0_ = concentration of DEX loaded into DEXHY.

### 4.8. In Vitro Evaluation of Tissue Adhesive Interactions of Dexketoprofen-Loaded Hydrogel

Bioadhesion studies were performed using TA.XT Plus ^®^texture analyzer (from Stable Micro Systems, Surrey, UK) in order to determine the force required for the detachment of HY and DEXHY from the biological membrane. A simulating membrane (cellulose membrane from a dialysis tubing 12 kDa) boiled and cooled beforehand was used as a substrate. A volume of 200 µL PBS at pH 7.2, T = 37 °C was used to simulate the biological fluid environment. The HY and DEXHY compressed hydrogels (ϕ = 8 mm) were attached to the mobile arm. The cellulose membrane and the samples were kept in contact for 60 s under 0.5 N contact strength. The detachment speed was set to 0.1 mm/s according to Hägerström et al. [74]. Two parameters were measured: detachment or peak force and work of adhesion by using the Exponent software (version 6.1).

### 4.9. In Vitro Cytotoxicity Studies

To evaluate the cytotoxic effects of DEXHY and HY hydrogels on mouse fibroblasts (3T3-L1 Cell Line Service, Appelheim, Germany), the in vitro MTT assay was used [75]. The primary purpose of cytotoxicity assessment was to calculate IC50 values.

#### 4.9.1. 3T3-L1 Cell Culture

The number of cells (3T3-L1 Cell Line Service, Appelheim, Germany) required in this study was calculated before cell cultivation (130 × 10,000 = 1,300,000 cells were needed since 10,000 cells/100 µL were desired in each well). The cells were grown in DMEM:F12 + 1% or 10% FBS +1% Pen-Strep. The culture was maintained at 37 °C, 5% CO_2_, and 100% humidity for 24 h.

#### 4.9.2. Cell Viability Assay

The effects of DEX, HY, and DEXHY on 3T3-L1 cell viability were assessed using a spectrophotometer with the 3-[4,5-dimethylthiazol-2-yl]-2,5-diphenyl tetrazolium bromide (MTT) assay. A medium control, a positive control (Triton-X, from Sigma-Aldrich, Hamburg, Germany), 7 different dose levels for the active substance, and 6 dose levels for 4 different formulations were used. At the end of 24 h, the old medium in the wells was aspirated and removed, and 100 µL of medium control, Triton-X, solutions containing pure active ingredient, and formulation samples were added to the relevant wells and incubated for 24 h. After 24 h of treatment, 50 μL of MTT solution was added to each well and then incubated in the dark for 4 h at 37 °C. At the end of 4 h, the medium in the wells was removed, and 150 µL of dimethyl sulfoxide (DMSO) was added to each well to dissolve the formed formazan crystals. Then, the surface of the plate was covered with aluminum foil and left in the plate shaker for 2 min to dissolve the crystals. Finally, the absorbance value in each well was measured at 570 nm using a BioTek Synergy H1a (BioTek Instruments, Winooski, VT, USA) microplate reader. The viability of the control group was accepted as 100%, and the viability of the other wells was calculated using Equation (4).

Cell viability = As/Ac × 100 (4)

where As and Ac refer to the absorbance values of the sample and control wells, respectively.

The following solutions were used during the experiments:

Complete medium (DMEM:F12 + 1% or 10% FBS +1% Pen-Strep): 45 mL of DMEM:F12 (obtained from Gibco Cat No: 11320033) + 5 mL of FBS (obtained from Gibco Cat No: 10500064) and 500 µL of Pen-Strep (obtained from Gibco Cat No: 15070063) in a sterile vessel.

Triton-X solution, 171 µM (positive control): 235 µL of stock Triton-X (obtained from Sigma-Aldrich Cat No: X100-100ML) solution was added onto 765 µL medium. Then, 1 µL of this was added to 999 µL medium. After that, 171 μL of intermediate stock was taken and added to 829 μL medium.

Sterile PBS solution: two PBS tablets (Thermo Fisher Scientific, Waltham, MA, USA) were dissolved in 400 mL water and then sterilized.

MTT solution was prepared at 5 mg/mL in PBS and sterilized by filtering through a 0.22 μm membrane filter in a biolaminar safety cabinet.

Dexketoprofen solutions: 10 mg of dexketoprofen (DEX) was weighed and dissolved in 10 mL medium.

Hydrogel solution: 1 mg of HY was weighed, added to 1 mL of complete medium, and placed in a 37 °C water bath. Then, solutions with concentrations of 500, 250, 125, 62.5, and 31.25 μg/mL were prepared from the previous solution.

Dexketoprofen-loaded hydrogel solution: 1 mg of DEXHY was weighed, added to 1 mL of complete medium, and placed in a 37 °C water bath. Then, solutions with concentrations of 500, 250, 125, 62.5, and 31.25 μg/mL were prepared from the previous solution.

### 4.10. Scratch Assay

Because cell migration is a crucial step for wound healing, the in vitro scratch test [76,77,78] was used to evaluate the therapeutic effect of HY, DEX, and DEXHY in cell migration capacity. Mouse fibroblasts (3T3-L1 cells line) (Cell Line Service, Appelheim, Germany) were used to test the in vitro regenerative effect. The 3T3-L1 cells suspended in DMEM medium with 1% FBS and 10% FBS were seeded in 24-well plates to grow in a monolayer for 48 h. The plates were cultured in a CO_2_ thermostat under standard conditions. Then, a sterile micropipette was held vertically to scratch the monolayer. The detached cells were removed with Hanks’ Balanced Salt Solution (HBSS) without Ca^2+^, Mg^2+^ (Thermo Scientific, Waltham, MA, USA). A filtered solution of DEX and freeze-dried hydrogels previously sterilized by exposure to UV radiation were added to the scratched cells layer. Monolayers of mouse fibroblasts with vertical wounds, cultured only in medium, served as controls. First, we assessed the influence of PBS concentration, and then we analyzed whether the rate of scratch closure changes compared to a control medium (cells with no hydrogels) and to each other. The scratch closure was monitored and imaged in 30 h intervals using Image J software v1.54 to assess the rate at which the neighboring cells filled in the damaged area. All scratch assays were performed in triplicate.

### 4.11. Statistical Analysis

Statistical evaluation was realized using one-way ANOVA and Tukey post hoc analysis. Differences between groups in terms of wound healing efficiency were considered statistically significant for *p* < 0.05 and were indicated with an asterisk (*). The significance level was further elaborated in the analyses using the following symbols: * *p* < 0.05, ** *p* < 0.01, *** *p* < 0.001, and **** *p* < 0.0001. All results were expressed as the mean of at least three experiments ± SD.

## 5. Conclusions

In this study, the obtained results demonstrate that the hydrogel based on alginate and poly(N-vinylcaprolactam) is a promising delivery system for the controlled topical application of DEX in order to support the normal wound healing process. This system comes with a series of advantages, such as prolonging the time of release and action, stimulating cell proliferation, and minimizing the specific side effects of NSAIDs. This study had some limitations because it was performed on animal cells, and our results might be different from those observed on human cells. However, we consider that the hydrogel loaded with dexketoprofen, a relatively new nonsteroidal anti-inflammatory drug with proven therapeutic efficiency, can be used for the care and healing of acute and chronic wounds, including postoperative ones, while benefiting from the analgesic and antipyretic action of dexketoprofen.

## Figures and Tables

**Table 1:** Pore size variations with hydrogel composition.

Hydrogel	Min. (µm)	Max. (µm)
HY	12.14 ± 6.15	86.05 ± 6.12
DEXHY	10.21 ± 7.08	70.22 ± 7.74

**Table 2:** Kinetic swelling parameters in PBS pH = 7.4 at 37 °C.

Sample	n	k (min^−1^)
HY	0.39	0.21
DEXHY	0.35	0.19

**Table 3:** Kinetic parameters of DEX release from DEXHY hydrogel.

Sample	Zero Order	First Order	Higuchi	Korsmeyer–Peppas		
	r^2^	r^2^	r^2^	r^2^	n	k
DEXHY	0.9678	0.9373	0.9455	0.9883	0.5336	0.21

r^2^ = correlation factor; n = diffusion exponent; k = release rate constant.

**Table 4:** Description of the hydrogels HY and DEXHY.

Hydrogel Code	Details
HY (AA-g-PNVCL)	Dexketoprofen-free hydrogel was obtained through ethanol precipitation followed by lyophilization of the polymeric matrix consisting of alginate grafted with poly(N-vinylcaprolactam) (PNVCL). That polymeric matrix resulted from the radical polymerization of NVCL and grafting of the formed PNVCL onto alginate through hydrogen bonding.
DEXHY (AA-g-PNVCL-DEX)	Dexketoprofen-loaded hydrogel obtained by the in situ loading of HY (AA-g-PNVCL) with dexketoprofen.

## Data Availability

The data presented in this study are available on request from the corresponding authors.
